# Expression of a novel surfactant protein gene is associated with sites of extrapulmonary respiration in a lungless salamander

**DOI:** 10.1098/rspb.2018.1589

**Published:** 2018-10-03

**Authors:** Zachary R. Lewis, Jorge A. Dorantes, James Hanken

**Affiliations:** Department of Organismic and Evolutionary Biology and Museum of Comparative Zoology, Harvard University, Cambridge, MA 02138, USA

**Keywords:** salamander, respiration, gene duplication, molecular evolution, surfactant, plethodontid

## Abstract

Numerous physiological and morphological adaptations were achieved during the transition to lungless respiration that accompanied evolutionary lung loss in plethodontid salamanders, including those that enable efficient gas exchange across extrapulmonary tissue. However, the molecular basis of these adaptations is unknown. Here, we show that lungless salamanders express in the larval integument and the adult buccopharynx—principal sites of respiratory gas exchange in these species—a novel paralogue of the gene *surfactant-associated protein C* (*SFTPC*), which is a critical component of pulmonary surfactant expressed exclusively in the lung in other vertebrates. The paralogous gene appears to be found only in salamanders, but, similar to *SFTPC*, in lunged salamanders it is expressed only in the lung. This heterotopic gene expression, combined with predictions from structural modelling and respiratory tissue ultrastructure, suggests that lungless salamanders may produce pulmonary surfactant-like secretions outside the lungs and that the novel paralogue of *SFTPC* might facilitate extrapulmonary respiration in the absence of lungs. Heterotopic expression of the *SFTPC* paralogue may have contributed to the remarkable evolutionary radiation of lungless salamanders, which account for more than two thirds of urodele species alive today.

## 1. Introduction

Most amphibians must confront the challenges of respiring both in water and on land. To do so, they use numerous gas exchange surfaces including the lungs, gills, integument, and buccopharyngeal mucosa, which are employed to varying extents depending on species and life-history stage. In adult salamanders, for example, the integument may be responsible for 50% or more of oxygen uptake [1,2]. Lability in sites of gas exchange is especially critical for metamorphosing species, which face different respiratory demands in air and water. Little is known, however, about the molecular mechanisms that enable the ontogenetic and evolutionary transitions from aquatic to aerial respiration. The mechanism of aerial respiration is even more enigmatic in lungless species, which rely entirely on extrapulmonary sites of respiration [1–3]. The family Plethodontidae includes more than two thirds of all living salamander species; most are fully terrestrial, and all adults lack lungs. Respiration takes place solely across the integument and buccopharyngeal mucosa, and also across the gills in aquatic larval forms, when present. Lunglessness is not unique to plethodontids—it has evolved several times in other amphibians, including salamanders, frogs and caecilians [4]—but its adaptive significance is unresolved [5,6].

How lungless salamanders are able to satisfy metabolic demands for oxygen is a topic of considerable interest. In theory, lunglessness limits thermal tolerance and maximum body size, yet lungless salamanders paradoxically occupy diverse thermal environments and attain relatively large body sizes. Plethodontids possess highly vascularized skin and buccopharyngeal mucosa, which may compensate for the loss of pulmonary respiration [1,7–9]. The buccopharyngeal membranes, in particular, may function as an adaptive respiratory surface that facilitates gas exchange, as evidenced by increased oscillation of the floor of the buccal cavity under hypoxia, high temperature or activity, which presumably serves to draw more air into the mouth [1,7,10]. Selection for efficient extrapulmonary respiration may have played a major role in the adaptive radiation of terrestrial plethodontids [1]. Indeed, the evolution of highly efficient cutaneous and buccopharyngeal respiration is believed to have freed plethodontids from the ontogenetic and functional constraints associated with the use of a buccal pump for pulmonary ventilation, thereby enabling them to occupy diverse habitats and evolve ballistic tongue projection [2,11,12].

To identify the molecular adaptations that might facilitate lungless respiration, we investigated the expression of a crucial pulmonary surfactant-associated protein in plethodontid salamanders. Proper lung function requires pulmonary surfactant, a complex and evolutionary variable mixture of molecules that facilitate mucus spreading and lung compliance and improve oxygen diffusion [13–15]. Surfactant-associated protein C (SFTPC) is a hydrophobic protein found in pulmonary surfactant that localizes to the lung's air–liquid interface. It reduces surface tension by aiding the adsorption and distribution of lipids within pulmonary surfactant and specifically enhances oxygen diffusion [14,16,17]. SFTPC also regulates production and turnover of phosphatidylcholine, a major component of pulmonary surfactant, and it limits the thickness of the hypophase, the liquid layer that lines the lung's inner surface [18–20]. Mucous layer thickness greatly impacts gas exchange between the environment and the blood supply [21]. Additionally, oxygen uptake is enhanced by the presence of surfactant in the hypophase, probably owing to convective effects that facilitate mixing of oxygen and mucus or increase the rate of oxygen trafficking [15,22]. The expression of SFTPC is highly conserved among tetrapods: all species evaluated previously express SFTPC exclusively in the lungs [23–25] (and electronic supplementary material, text).

## 2. Results and discussion

Despite lacking lungs as adults, plethodontid salamanders express gene transcripts with high sequence similarity to *SFTPC* (electronic supplementary material, figure S1). Owing to the consistent restriction of *SFTPC* expression to the lungs and to SFTPC's conserved role across tetrapods, we compared *SFTPC* expression between lungless and lunged salamanders. Surprisingly, several species of salamanders express two transcripts with high sequence identity to annotated *SFTPC* sequences (electronic supplementary material, figure S1a,b). Both transcripts exclusively match SFTPC within the NCBI nucleotide collection database, but one possesses higher sequence similarity to amniote and frog *SFTPC*. We denote the transcript with lower similarity *SFTPC*-*like*. Phylogenetic analyses support the hypothesis that *SFTPC*-*like* represents a previously undescribed salamander-specific paralogue of the highly conserved lung-specific gene, *SFTPC* (electronic supplementary material, figures S1, S2 and S4, and text). However, gene trees for *SFTPC* and *SFTPC*-*like* are not fully resolved, and there remains the possibility that SFTPC-like was present in the most recent common ancestor of sarcopterygians and subsequently lost in several lineages including lungfish, frogs and amniotes (see the electronic supplementary material, text). SFTPC-like may maintain or partially maintain the characteristic hydrophobic α-helical configuration of the SFTPC mature peptide [16] (electronic supplementary material, figure S1c).

**Figure 1.**
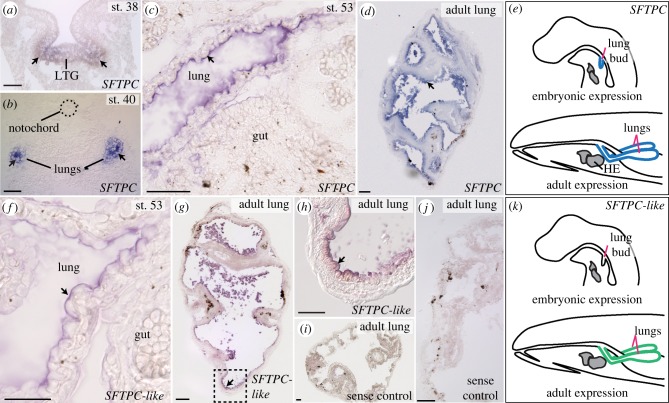
Expression patterns of *SFTPC* and *SFTPC*-*like* in a lunged salamander, *Ambystoma mexicanum*, visualized with antisense whole-mount ISH. Arrows point to representative regions of expression. *SFTPC* is expressed in the embryonic laryngotracheal groove (LTG) (*a*) or lungs (*b–d*) of all stages examined between embryo (st. 40), juvenile (st. 53) and adult. (*f–h*) *SFTPC*-*like* is also expressed specifically in the lung in juveniles and adults, but at a lower level than *SFTPC*; it is not expressed in embryos. (*h*) Boxed region in (*g*). (*i,j*) Negative (sense) control runs in parallel with *SFTPC*-*like*. The dark brown and black cells are melanophores (*e*,*k*). Schematic sagittal views summarize the expression sites of *SFTPC* and *SFTPC*-*like*. Blue regions denote high expression; green indicates lower level. (*a*,*b*,*d*) and (*g–j*) depict transverse sections; (*c*) and (*f*) are sagittal sections, anterior to the left. Scale bars: 100 µm. Additional abbreviation: HE, heart.

Numerous studies of tetrapods localize SFTPC exclusively to the lungs [23–25]. *SFTPC* and *SFTPC*-*like* in the lunged salamander *Ambystoma mexicanum* match this highly conserved pattern (figure 1). As visualized by *in situ* hybridization (ISH), *SFTPC* and *SFTPC*-*like* transcripts are observed only in the alveolar epithelial cells lining the lungs and trachea (figure 1; electronic supplementary material, figure S6). The alveolar epithelial cells are extremely squamous, often with a height of less than 200 nm (electronic supplementary material, figure S7), which causes the expression pattern to appear restricted to the cell surface adjacent to the lumen of the lung. Yet, both *SFTPC* and *SFTPC*-*like* are expressed throughout the epithelial cytoplasm. *SFTPC*-*like* expression, however, is lower than *SFTPC* and the genes are expressed at different times: *SFTPC*-*like* is low or non-existent before hatching, whereas *SFTPC* is expressed in embryos beginning immediately following the formation of the laryngotracheal groove, a ventral outpocketing of the foregut that precedes lung outgrowth (figure 1*a*,*b*), and continuing into adulthood (figure 1*d*).

**Figure 2.**
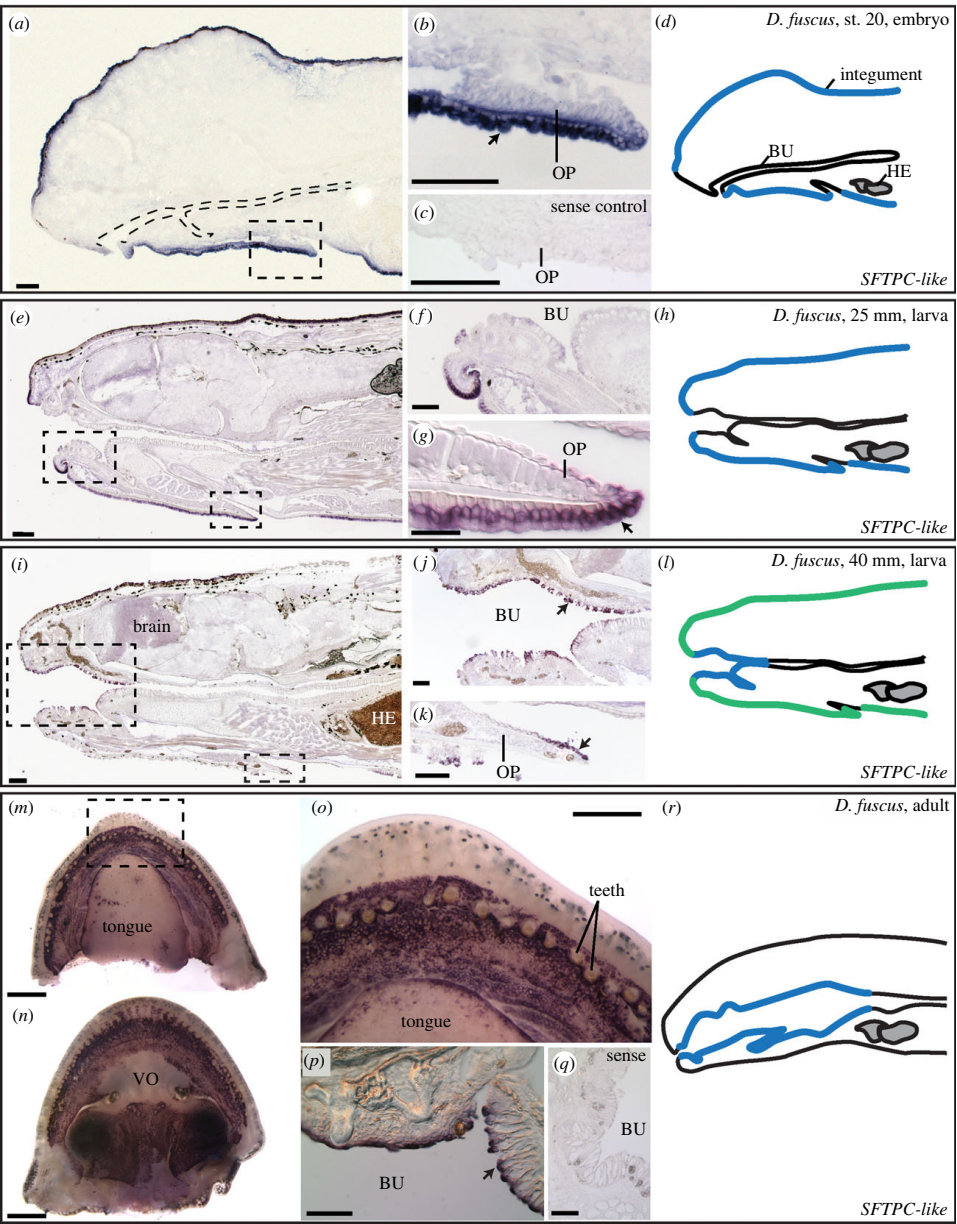
Expression of *SFTPC*-*like* in the lungless salamander *Desmognathus fuscus*, visualized with antisense whole-mount ISH. Arrows point to representative regions of expression. (*a–c*) In embryos, *SFTPC*-*like* is expressed in the integument. (*b*) Boxed region in (*a*). (*c*) Negative (sense) control. (*e–g*) *SFTPC*-*like* is expressed in the larval integument, 25 mm total length. (*i–k*) *SFTPC*-*like* in a larva just prior to metamorphosis, 40 mm total length. Expression has declined in the integument but is now present in the buccopharyngeal mucosa (BU). (*m–q*) Adult expression of *SFTPC*-*like* is confined to the buccopharyngeal cavity. (*d*,*h*,*l*,*r*) Schematic sagittal views summarize the expression sites of *SFTPC*-*like*. Blue regions correspond to high expression; green indicates lower level. The dark black cells in 25 mm, 40 mm and adult stages are melanophores. (*a–l*) Sagittal sections, anterior to the left; (*p*,*q*) transverse sections. Scale bars: (*a–c*,*e*,*f*,*i–k*,*p*,*q*), 100 µm; (*g*) 50 µm; (*m*,*n*) 1 mm; (*o*) 0.5 mm. Additional abbreviations: HE, heart; OP, opercular covering; VO, vomer.

By contrast, *SFTPC*-*like* is expressed dynamically in lungless salamanders. In embryos and early larvae of *Desmognathus fuscus*, a metamorphosing species, *SFTPC*-*like* is expressed throughout much of the integument, with reduced staining on the dorsal (internal) surface of the operculum (gill covering) and in the limbs (figure 2*a–h*). Although the larval gills are important respiratory sites [26], we observed no expression of *SFTPC*-*like* in them by ISH. Expression begins to diminish in the integument immediately before metamorphosis, but at the same time, it expands to the buccopharyngeal mucosa (oral epithelium) (figure 2*i–l*). Integumentary expression at this stage is patchy: remaining *SFTPC*-*like*-positive cells are displaced towards the apical surface and display an irregular morphology (figure 2*i*,*k*). Cessation of integumentary expression of *SFTPC*-*like* coincides with several metamorphic transitions, but especially moulting [27], when the integument is profoundly remodelled from a simple stratified epithelium to a thickened pseudostratified tissue rich in acinous glands (figure 2*i*,*k*; electronic supplementary material, figure S5).

Immediately following metamorphosis, expression is absent from the integument and restricted to the buccopharyngeal mucosa (figure 2*m–r*). In adults, *SFTPC*-*like* is expressed in the buccal cavity and adjacent pharynx (figure 2*m–r*): it is confined to oral mucosa, with a strict boundary at the marginal teeth. *SFTPC*-*like* is not expressed along the vomerine bones (near the internal nares) or in the dorsal midline of the tongue, although it is strongly expressed along the tongue margin (figure 2*m*,*o*).

Unlike *SFTPC*-*like*, *SFTPC* is expressed at an extremely low level in embryos of lungless species, which develop a transient lung rudiment [28,29]. The *SFTPC* transcript detected from transcriptome sequencing of the lung rudiment of *Plethodon cinereus* (electronic supplementary material, data file S1) could not be cloned from either *P. cinereus* or *D. fuscus*, nor was it found in adult plethodontid transcriptomes. This suggests that *SFTPC* expression is inhibited by lung loss in lungless species, with the exception of *SFTPC* expression at certain embryonic stages in the presumptive lung region.

**Figure 3.**
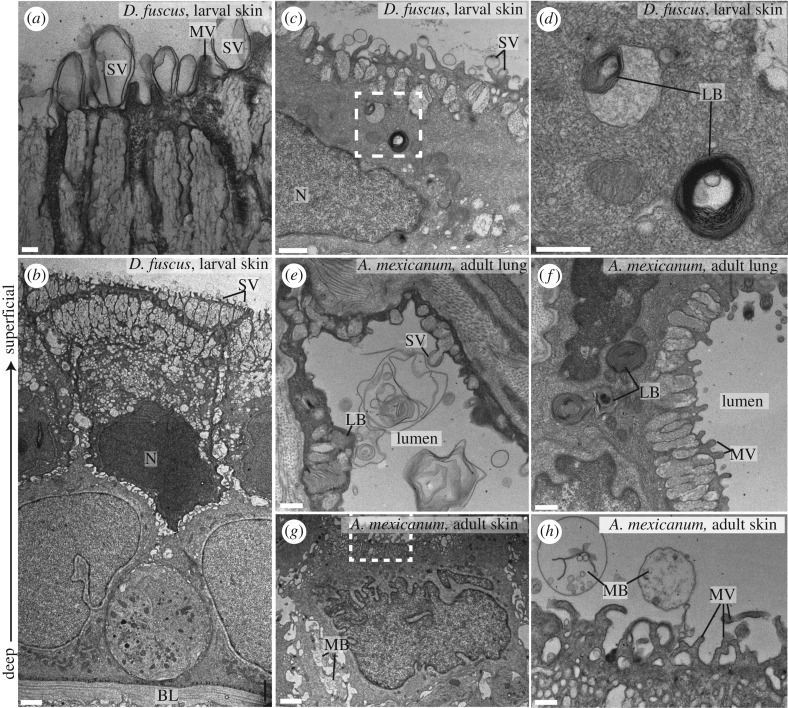
Secretory activity and lamellar bodies in the larval integument of lungless *Desmognathus fuscus* resemble those in the lung of *Ambystoma mexicanum*. (*a–d*) Transmission electron micrographs of a 24 mm *D. fuscus*. (*a*) The superficial (apical) surface is covered with secretory vesicles (SV), which emerge from columnar vacuolar structures, and is interspersed with microvilli (MV). (*b*) Sagittal section through the epidermis; the superficial surface points upwards. (*c*,*d*) Lamellar bodies (LBs), indicative of surfactant production, are visible in the integument. The boxed region in (*c*) is enlarged in (*d*). (*e*,*f*) Lamellar bodies and secretory vesicles in the distal portion of the lung of an adult *A. mexicanum*; transverse section. (*g*,*h*) Transverse section of gular integument of adult *A. mexicanum*. The boxed region in (*g*) is enlarged in (*h*). Multivesicular bodies (MBs) are visible in extracellular spaces in (*g*) and external to the integument in (*h*), but there is no indication of active secretion of vesicles. The integument does not play a pronounced secretory role in this species. Additional abbreviations: BL, basal lamina; N, nucleus. Scale bars: (*a*) 200 nm; (*b*,*g*) 2 µm; (*c*) 1 µm; (*d*,*e*,*f*,*h*) 500 nm.

The larval integument of *D. fuscus* displays pronounced secretory activity (figure 3*a–d*). The outer layer—the stratum corneum—is a protective layer composed of anuclear keratinized cells. It displays an extremely high level of secretory activity evidenced by the near universal distribution of bilamellar secretory vesicles along its superficial surface (figure 3*a*, ‘SV’). Just basal to the stratum corneum is a layer of secretory cells, which are heavily vacuolated at their apical extent. The stratum corneum and the layer of secretory cells are syncytial, but a dark and consistent division appears between them (figure 3*b*). Bilamellar secretory vesicles virtually identical to those observed on the stratum corneum of *D. fuscus* are secreted from alveolar epithelial cells in the lung of *A. mexicanum* but are not present in its integument (figure 3*e*,*g*,*h*; electronic supplementary material, figure S7, ‘SV’).

Pulmonary surfactant is trafficked in lamellar bodies in alveolar epithelial cells. These lamellar bodies are distinct from other ultrastructural lamellar elements in that they are large in diameter and spherical in shape [30–32]. Furthermore, these lamellar bodies are distinct from the bilamellar structures identified on the surface of the integument, in that they have many lamellae. The larval integument of *D. fuscus* contains large (0.5–0.75 µm diameter), spherical lamellar bodies (figure 3*c*,*d*, ‘LB’; electronic supplementary material, figure S8) that closely resemble those found in alveolar epithelial cells, which otherwise are known only from the lungs of other tetrapods. The packaging and secretion of surfactant via lamellar bodies relies on a number of proteins including members of the ATP-binding cassette (ABC) transporter superfamily. Lamellar bodies also characteristically contain lysosomal enzymes such as Lamp3 [33]. It is unknown whether these genes are expressed in the *D. fuscus* integument. Interestingly, *SFTPC*, *ABCA3* and *Lamp3* are all regulated by the gene Nkx2.1 in mice [34–36], offering a potential mechanism whereby heterotopic expression of one gene could drive the machinery for production of lamellar bodies outside the lungs. Secretory activity and the presence of these distinctive lamellar bodies strongly suggest that *D. fuscus* produces surfactant in extrapulmonary sites of gas exchange, which correspond to sites of *SFTPC*-*like* expression. However, additional data from immunohistochemistry and mass spectroscopy are needed to determine if *SFTPC*-*like* transcripts are translated and whether this protein is then processed and secreted in a similar fashion to SFTPC in the lung.

Despite the lung- and trachea-specific expression of *SFTPC* and *SFTPC*-*like* in *A. mexicanum* revealed by ISH and the numerous reports of lung-specific expression of *SFTPC* in mammals and amphibians (electronic supplementary material, text), recently published transcriptomes of *A. mexicanum* purport to map low numbers of *SFTPC* and *SFTPC*-*like* reads to several tissues, including blood vessels, bone, heart, regenerating limbs and mixed stages of whole embryos [37]. Therefore, *SFTPC* and *SFTPC*-*like* may not be entirely lung-specific transcripts in lunged salamanders. Additional studies are needed, however, to evaluate the alternative interpretation that contamination, false index assignment [38]**,** mapping, or assembly issues might have yielded spuriously mapped reads.

Passive diffusion across a tissue layer, as occurs during cutaneous respiration, is a function of several variables. These include the size (area) of the surface over which gas exchange occurs and the thickness of the barrier between the underlying blood supply and the environment [3]. Barrier thickness depends mainly on the distance between the environment and the blood supply, but also on the thickness of the mucous layer between the environment and respiratory tissue. Diffusivity of mucus is about 30% lower than water [21]. Reduction of surface tension by pulmonary surfactant helps maintain a thin layer of airway surface liquid [20] and increases convection within the mucus, which together result in increased oxygen uptake [22]. SFTPC indirectly influences mucous layer thickness in the lung by regulating the production of phosphatidylcholine [18]. Pulmonary surfactant aids oxygen transport across the air–liquid interface, and hydrophobic surfactant proteins increase the rate of oxygen diffusion twofold over surfactant lipid alone [15,17]. If SFTPC-like facilitates cutaneous respiration, then it may do so through a reduction of effective barrier thickness or an increase in diffusivity of the mucous layer. Pulmonary surfactant also plays non-respiratory roles, including facilitation of mucus spreading, innate immune defence, anti-oedema agent, hydrostatic gas exchange and preventing adhesion of lung surfaces when the lungs deflate [13,39,40]. It is possible that extrapulmonary surfactant produced in *D. fuscus* is performing one or more of these functions instead of, or in addition to, facilitating gas exchange.

At present, any functional role for the gene *SFTPC*-*like* is hypothetical. It remains to be determined if the gene product is produced, and whether and where it is secreted. Therefore, our proposals regarding the protein and its possible activity in plethodontid respiration are preliminary. Nevertheless, spatial and temporal expression of *SFTPC*-*like* and the ultrastructure of the associated integument suggest that, following *SFTPC*-gene duplication, *SFTPC*-*like* became neofunctionalized for extrapulmonary respiration in lungless salamanders. Sequence and structural conservation of *SFTPC*-*like* and *SFTPC* suggests that both proteins function similarly (electronic supplementary material, figure S1). However, to confirm neofunctionalization requires a more detailed functional characterization of SFTPC-like. This may include proteomic characterization of skin and buccopharyngeal secretions, and assessment of whether SFTPC-like displays surface activity or aids gas exchange. Further studies are also needed to focus on whether the *SFTPC*-*like* transcript is translated, and to characterize the dynamics of *SFTPC*-like trafficking and secretion, if it is produced.

Future work may also include determining with increased phylogenetic and technical precision how expression of *SFTPC*-*like* has evolved in salamanders. For instance, although the transcript is present in all surveyed salamander transcriptomes (electronic supplementary material, figure S1), it is not known where this transcript is expressed in other species of lungless salamanders besides *D. fuscus*. Moreover, we have considered only one of four known lineages of lungless amphibians. We posit that *SFTPC* was duplicated in salamanders (electronic supplementary material, figures S1, S2 and S4). However, additional genomic data for caecilians and frogs promise to bring the evolution of SFTPC-like into greater focus. It is unlikely that duplication of *SFTPC* is a prerequisite for lung loss, which has convergently evolved at least two times outside of salamanders, and because many amphibians rely primarily on non-pulmonary respiration [3].

In metamorphosing species such as *D. fuscus*, respiratory and fluid retention demands shift dramatically upon the transition from aquatic to terrestrial habitats [1]. Cutaneous water loss becomes a critical liability, but reduced skin permeability hinders cutaneous gas exchange. To counter this limitation, terrestrial plethodontids show increased reliance on buccopharyngeal respiration [1]. Ontogenetic shift of *SFTPC*-*like* from the larval integument to the adult buccopharyngeal cavity during metamorphosis (figure 2) correlates with the transition from aquatic to aerial respiration; *SFTPC*-*like* is expressed at the preferential sites of gas exchange at each life-history stage [1]. However, it is also possible that instead of playing a direct role in facilitating gas exchange, extrapulmonary surfactant balances fluid retention and respiratory demands by aiding fluid uptake from mucus. Such a role would be consistent with the proposed anti-oedema properties of surfactant within the lungs [13]. Finally, the absence of *SFTPC*-*like* expression in the gills, important sites of larval respiration, remains enigmatic and uninvestigated. If SFTPC-like functions to aid respiration, then presumably its expression in the gills would be both beneficial and expected.

Gene duplication is increasingly recognized as a driving force of evolutionary innovation [41]. While gene duplication does not always lead to functional divergence [42,43], regulatory changes in duplicated genes, such as altered expression sites, may enable the evolution of novel traits in individual lineages. Many studies of the evolutionary phenomenon of adaptive radiation have emphasized morphological traits whose appearance in particular lineages promote phylogenetic and ecological diversification [44]. We propose that such morphological traits, or key adaptations, work in concert with novel and functionally significant molecular features to enhance evolutionary success, and that such instances of concerted evolution are more widespread than is currently recognized.

In plethodontid salamanders, it is possible that the combination of morphological adaptations [7,9,45] and deployment of a novel surfactant protein enable efficient extrapulmonary respiration via the buccopharynx and integument. Conserved expression of *SFTPC*-*like* in lunged salamanders relative to *SFTPC* may be owing to dosage-sharing between SFTPC and SFTPC-like, which constrains tolerable mutations in *SFTPC*-*like* gene regulation [42]. Indeed, lung loss may have resulted in relaxed stabilizing selection for SFTPC-like gene regulation, thereby enabling the evolution of novel expression patterns. A greater understanding of the evolution and function of SFTPC-like in additional salamander species will yield a more complete picture of the evolution and consequences of lung loss, while functional studies of SFTPC-like promise to reveal whether it plays similar roles to SFTPC or has potential therapeutic applications. Given the convergent evolution of lung loss in several amphibian lineages, it will be interesting to investigate the molecular physiology of other lungless taxa. Neofunctionalization of SFTPC-like may represent an additional mechanism by which plethodontid salamanders have become one of the most speciose and geographically widespread clades of vertebrates on Earth, despite the theoretical limitations on thermal tolerance and body size imposed by lunglessness.

## 3. Material and methods

### (a) Animal husbandry

*Desmognathus fuscus* (northern dusky salamander) embryos were field collected from the following two localities under Massachusetts Department of Fish and Wildlife collection permits 080.11SCRA (2012), 027.13SCRA (2013), 083.14SCRA (2014) and 022.15SCRA (2015) and appropriate local permits: Ashfield, Mass. (42.483111, −72.761263) and Mass Audubon Wachusett Meadows Preserve (42.450922, −71.913009). Adults were collected from Willard Brook State Forest (42.671606, −71.776156). *Plethodon cinereus* (eastern red-backed salamander) embryos were field collected from Willard Brook State Forest (42.671606, −71.776156). *Desmognathus fuscus* embryos were maintained at 15**°**C in 0.1× Marc's modified Ringer solution (MMR; 0.01 M NaCl, 0.2 mM KCl, 0.1 mM MgSO_4_, 0.2 mM CaCl_2_, 0.5 mM HEPES, pH 7.4). Following hatching, larvae were fed *Artemia* spp. and maintained at 17–20**°**C until they metamorphosed at approximately seven months post-hatching. Older larvae were hand-fed blood worms. Embryos and larvae were sampled at intermediate stages from embryogenesis until 3–5 days post-metamorphosis and fixed overnight in MEMFA (0.1 M MOPS (pH 7.4), 2 mM EGTA, 1 mM MgSO_4_ and 3.7% formaldehyde) at 4**°**C, then dehydrated and stored at −20**°**C in 100% methanol. Adults were fixed in a similar manner immediately upon collection.

*Ambystoma mexicanum* (Mexican axolotl) embryos were obtained from the *Ambystoma* Genetic Stock Center, University of Kentucky, and maintained in 20% Holtfreter solution at 17**°**C. Larvae were raised similarly to larval *D. fuscus*. Fixation was performed as described above.

*Ambystoma mexicanum* were staged according to Bordzilovskaya *et al.* [46] and Nye *et al.* [47]. *Desmognathus fuscus* embryos were staged using a staging table derived for *P. cinereus*, as their developmental timing and morphology are grossly similar [48].

### (b) Polymerase chain reaction

Embryonic cDNA from *D. fuscus*, *P. cinereus* and *A. mexicanum* was used to clone the gene *SFTPC-like*. RNA was isolated from homogenates of whole animals at a variety of embryonic stages using TRIzol Reagent (Invitrogen/Thermo Fisher Scientific, Grand Island, NY) and reverse-transcribed to cDNA using iScript reverse transcriptase (BioRad, Hercules, CA, USA). The gene *SFTPC* was cloned from *A. mexicanum* and repeated attempts were made to clone *SFTPC* from plethodontids. Degenerate and non-degenerate polymerase chain reaction (PCR) primers were used (electronic supplementary material, table S1).

Primers were designed based on alignment of *SFTPC* sequences from *D. fuscus*, *Xenopus laevis*, *Xenopus tropicalis*, *Anolis carolinensis*, *Neovison vison*, *Bos taurus*, *Monodelphis domesticus* and *Homo sapiens*. All sequences but one were obtained from GenBank; the *D. fuscus* sequence was kindly provided by Dr David Weisrock.

### (c) Transcriptome assembly

Transcriptomes for *P. cinereus* and *A. mexicanum* were prepared from microdissected tissue from pharyngeal endoderm and mesoderm of embryos and from the lung of a juvenile *A. mexicanum*. Total RNA was used for library preparation by using the IntegenX PrepX RNA-Seq Library Kit (IntegenX, Pleasanton, CA, USA) on an Apollo 324 robotic sample preparation system (WaferGen Biosystems, Fremont, CA, USA), closely following kit instructions. Agencourt Ampure XP beads were used for magnetic purification steps (Beckman Coulter, Indianapolis, IN, USA). Beads were kept at room temperature for 15 min before starting block set-up. Beads were added last to the block, after a 30 s vortex to fully resuspend them.

Following cDNA synthesis, the concentration of samples was assessed by using a Qubit 1.0 fluorometer (Invitrogen/Thermo Fisher Scientific, Grand Island, NY, USA), high-sensitivity dsDNA reagents (Molecular Probes/Thermo Fisher Scientific, Grand Island, NY, USA) and Qubit Assay Tubes (Invitrogen). Samples were diluted to 20 µg ml^−1^ and then sheared on a Covaris S220 Focused ultrasonicator (Covaris, Woburn, MA, USA) using a 72-s protocol and targeting 220/320 bp output. TapeStation HS D1 K tape (Agilent) was used to examine sheared DNA for optimal size range. BIOO Scientific NEXTflex DNA barcodes (no. 514102, Austin, TX, USA) were diluted to 5 µm and used in the IntegenX PrepX DNA Library ILM prep kit (no. P003279, Pleasanton, CA, USA). Library prep was performed on the Apollo 324 using the kit manufacturer's precise instructions. Aliquots (5 µl) of approximately 2-µg ml^−1^ samples were then subjected to four PCR amplification cycles using NEB Next Master Mix (no. M0541S, NEB, Ipswich, MA, USA) and NEXTflex Primer Mix (BIOO Scientific) and the following cycle conditions: denaturation at 98**°**C for 120 s; 5 cycles of 98**°**C for 30 s, 6°C for 30 s and 72**°**C for 60 s; and final extension for 5 min at 72**°**C. The Agilent Apollo 324 was used for cleanup of PCR samples using the built-in PCR cleanup protocol and Agencourt Ampure XP beads. Libraries were analysed with Qubit 1.0, TapeStation and quantitative PCR (qPCR) to assess library concentration, size and quality. Samples were each diluted to 0.29 nM concentration and then pooled. Two lanes of 2 × 150 bp Illumina HiSeq 2500 Rapid Run RNA-sequencing (Illumina, San Diego, CA, USA) yielded a total 232.4 reads that passed filter.

Sequenced reads were trimmed with Trimmomatic [49] and concatenated. Ribosomal rRNA reads were removed by using Bowtie [50] and a custom database of known rRNA sequences for each species. Transcriptomes were assembled *de novo* using Trinity [51,52]. BLAST databases were created from the *de novo* assemblies and used to identify *SFTPC* and *SFTPC-like* sequences.

### (d) Surfactant-associated protein C phylogeny

*SFTPC* sequences were identified through BLASTX, TBLASTN and TBLASTX searches of sequenced transcriptomes from this study (*P. cinereus* and *A. mexicanum*) as well as from the transcriptomes listed in the electronic supplementary material, table S2.

Sequence identifiers and corresponding sequence data are provided in the electronic supplementary material, data file S1.

*SFTPC* sequences from annotated genomes were also taken from NCBI and ENSEMBL (electronic supplementary material, data file S1). Outgroup proteins were selected based on previous phylogenies of SFTPC [53,54]. Predicted amino acid sequences were generated from all nucleotide sequences. Multiple sequence alignment was performed using PRANK [55]; resulting alignments were visually inspected (electronic supplementary material, data file S2). ProtTest was used to identify an appropriate amino acid substitution model [56]. The optimal amino acid substitution model was JTT + G [57], as judged by Akaike information criterion. Subsequently, 95% maximum clade credibility gene trees were reconstructed in MrBayes (v. 3.2.6) [58] using Markov chain Monte Carlo analysis with one million generations sampled every 100 generations and a relative burn-in of 25%. Convergence of the posterior probabilities was assessed by examining output statistics, including the potential scale reduction factor, which equalled or exceeded 1.000.

A phylogeny was also constructed in RAxML (v. 8.2.10) [59] by using 1000 bootstrap replicates and the aforementioned amino acid substitution model (electronic supplementary material, figure S2). Tree topology was largely concordant with the Bayesian tree generated in MrBayes, the sole exception being support for placement of the SFTPC homologue from the coelacanth, *Latimeria chalumnae* (see the electronic supplementary material, text).

JalView was used to generate the multiple sequence alignment image [60].

PHYLDOG [61] was used to estimate gene duplication of SFTPC. A guide tree was constructed using NCBI taxonomy for major groups and [62] for amphibian relationships (electronic supplementary material, figure S3). Topology optimization was not used.

### (e) *In situ* hybridization

Embryos were fixed overnight in 4% paraformaldehyde (PFA) or MEMFA at 4**°**C, dehydrated and stored in 70 or 100% MeOH at −20**°**C. Whole-mount mRNA ISH was performed by rehydrating samples, which were then treated with 5–10 µg ml^−1^ proteinase K for 30–60 min, washed with PBTw (137 mM NaCl, 2.7 mM KCl, 10 mM Na_2_HPO_4_, 1.8 mM KH_2_PO_4_ and 0.2% Tween-20), post-fixed in 4% PFA, washed with PBTw and pre-hybridized in hybridization buffer for 2 h at 65**°**C (hybridization buffer: 50% formamide, 5× SSC, 0.1 mg ml^−1^ heparin, 1× Denhardt solution, 0.01% CHAPS, 0.2 mg ml^−1^ tRNA and 0.1% Tween-20; all solutions were RNase-free). DIG-labelled riboprobes were diluted approximately 1 : 40 in hybridization buffer, denatured at 85**°**C for 10 min and then added to specimens. Hybridization was carried out overnight at 65**°**C. Post-hybridization washes were performed with a solution of 50% formamide, 5× SSC and 0.2% Tween-20 at 65**°**C for eight changes of 30 min each. Specimens were washed with maleic acid buffer plus 0.2% Tween-20 (MABT) prior to blocking and antibody incubation. Antibody block solution included 20% heat-inactivated sheep serum and 2% blocking reagent (Roche, Penzberg, Germany) in MABT. Samples were incubated overnight at 4**°**C with 1 : 2500 anti-DIG-AP Fab fragments (Roche) diluted in blocking solution. Extensive washes with MABT were performed prior to colour development using BM-Purple (Roche) or NBT/BCIP (Sigma, St Louis, MO, USA). Colour development occurred over several hours. Embryos were then embedded for cryosectioning at 14–16 µm thickness. Photographs were taken using a Leica DMRE microscope (Wetzlar, Germany) equipped with a QImaging Retiga 2000r camera and a QImaging RGB slider (Model: RGB-HM-S-IR; Surrey, Canada) and Volocity 6.0 software (PerkinElmer, Waltham, MA, USA).

### (f) Structure models

An experimentally determined protein data bank (PDB) model for porcine SFTPC [16] was downloaded from the Research Collaboratory for Structural Bioinformatics PDB. The secondary and supersecondary structure of *D. fuscus* SFTPC-like was predicted using Quark *Ab initio* Protein Structure Prediction [63]. The N- and C-terminal extent of the *Desmognathus* SFTPC-like sequence was chosen based on alignment with mature forms of SFTPC found in mammals. Protein structure prediction was also performed with I-TASSER [64].

A structure model for *D. fuscus* SFTPC-like was also predicted in SWISS-MODEL [65] using the molecular structure derived by Johansson *et al*. [16] as a template. The PDB files for each SFTPC-like model were imported into PyMol (Schrödinger) and aligned with the SFTPC model to graphically illustrate structural similarities.

### (g) Transmission electron microscopy

Two 24 mm (total length) *D. fuscus* larvae were euthanized and decapitated. Specimens were then dissected in fixative (2.5% glutaraldehyde and 2% paraformaldehyde in 0.1 M HEPES; the aldehydes were free of alcohol stabilizers). The head was cut into three 1 mm sagittal sections. An 18 cm adult *A. mexicanum* was euthanized and then dissected in fixative. Samples of the gular integument from the ventral head, the oral epithelium and the lungs were trimmed to 1 mm thick pieces in fixative and fixed as above.

The samples were left in fixative for 3 days and then washed twice quickly with 0.1 M HEPES and three times for 5 min each with Milli-Q H_2_O (mqH_2_O). Next, samples were fixed for 24 h at 4**°**C in aqueous 1% osmium tetroxide, followed by five washes in mqH_2_O for 5 min each. Subsequently, specimens were stained with 2% uranyl acetate (EMS, Hatfield, PA, USA) overnight at 4**°**C, then washed two times for 5 min each with mqH_2_O. Specimens were dehydrated with 5 min washes of 50, 70 and 95% ethanol, followed by three 10 min washes with 100% ethanol, then two quick rinses with propylene oxide (PO). Specimens were embedded in Embed 812 resin (EMS) formulated to medium hardness by rinsing 30 min each in 1 : 1 PO to Embed 812, 1 : 2 PO to Embed 812, then 60 min in 1 : 4 PO to Embed 812. Specimens were then transferred to 100% Embed 812 and incubated overnight at room temperature, followed by two subsequent changes of Embed 812, over a total embedding time of 48 h. Samples were then positioned in moulds and placed at 60**°**C for 3 days to polymerize.

Sectioning was performed on a Leica UCT ultramicrotome, using glass knives for trimming blocks and generating semi-thin (1 µm) sections, and a DiATOME diamond knife for generating thin sections of approximately 60–100 nm thickness (target thickness: 80 nm). Sections were flattened with chloroform vapour, transferred onto precoated Formvar/carbon 200 mesh copper grids (no. 01803F, Ted Pella, Redding, CA, USA) and dried on filter paper.

Grids were imaged with an FEI Tecnai G2 series F20 transmission electron microscope (TEM; Hillsboro, OR, USA) at 80 kV using a Gatan CCD camera and Gatan Digital Micrograph Software (Pleasanton, CA, USA).

## Supplementary Material

Supplemental text and figures

## Supplementary Material

Supplemental Data File 1

## Supplementary Material

Supplemental Data File 2

## Data accessibility

All data are available as electronic supplementary material, data files S1 and S2.
