# Supplementary material for: Expression of a novel surfactant protein gene is associated with sites of extrapulmonary respiration in a lungless salamander
Source: Proc Biol Sci. 2018 Oct 3;285(1888):20181589. doi: 10.1098/rspb.2018.1589 (PMC6191699; doi:10.1098/rspb.2018.1589)
Supplement: Supplemental text and figures [file rspb20181589supp1.pdf]

Supporting Information for

Expression of a novel surfactant protein gene is associated with sites of extrapulmonary respiration in a lungless salamander

Zachary R. Lewis, Jorge A. Dorantes, James Hanken

correspondence to: zrlewis@gmail.com

This PDF file includes:

Supplemental Text
Tables S1 – S2
Figures S1 – S8
Captions for data files S1 and S2
References for supporting text and figures

Other Supplementary Materials for this manuscript includes the following:

Supplemental Data File 1
Supplemental Data File 2

Supplemental Text

SFTPC expression specificity

The expression pattern of SFTPC is highly conserved: all tetrapods express SFTPC exclusively in the lungs [1–9]. In anamniotes, SFTPC is expressed throughout the lung [5,6,10], whereas in mammals it is confined to alveolar type II cells. Four reports cite expression of SFTPC outside of the lungs in humans, but each report has methodological problems, including possible contamination, that may make such claims unreliable. Mo et al. (2006) report SFTPC in human fetal and adult skin [11]. This claim, however, relies on immunohistochemical data obtained with an antibody that may yield spurious labeling, and on RT-PCR data that was not followed up with sequencing. RT-PCR is subject to contamination and mispriming. Bräuer et al. (2009) report SFTPC expression in submandibular and parotid glands based on RT-PCR and western immunoblots [12]. The western blots reveal a protein of the expected size of the SFTPC pro-protein, and RT-PCR of focal cDNA is performed alongside a positive control, but there is no follow-up sequencing. Schicht et al. (2015) report SFTPC in saliva from human patients based on western blots and ELISA [13]. The antibody used to detect SFTPC is not identified, however, and the isolated band is at 16 kDa while SFTPC proprotein is 21 kDa and mature SFTPC 3.7 kDa [14]. Additionally, saliva may be subject to contamination by surfactant produced in the lungs. Finally, Schob et al. (2013) report SFTPC expression in central nervous system tissue and cerebrospinal fluid based on RT-PCR, but they fail to rule out the possibility of genomic DNA contamination [15]. Their western blots also fail to demonstrate a band at the expected size for SFTPC given the antibody they employed, and the authors express confusion about how mRNA for SFTPC could be present in cerebrospinal fluid. In sum, there are problems with all recent studies that cite extrapulmonary expression of SFTPC in humans. At the same time, numerous studies in mammals and frogs, including ISH and reporter knockins, have failed to demonstrate extrapulmonary expression of SFTPC. Nevertheless, it remains possible that humans and perhaps other animals endogenously express SFTPC outside of the lungs. Optimally, *in situ* hybridization and transcriptome sequencing should be used to validate the human results presented above.

Evidence for Duplication of SFTPC

SFTPC and SFTPC-like sequences diverge from one another within exonic regions, but not according to putative splice boundaries (Fig. S1a), which indicates that SFTPC-like is not an isoform of SFTPC. While SFTPC-like is divergent from SFTPC sequences, it is not an ortholog of a closely related BRICHOS domain-containing gene (Fig. S1b). We found SFTPC-like expressed in eleven species of lunged and lungless salamanders (Fig. S1a, b; Supplemental Data File 1); most also express SFTPC.

In Bayesian and maximum likelihood gene trees for SFTPC sequences from amphibians, amniotes, lungfish and coelacanth, tetrapod SFTPC and SFTPC-like fall into well-supported monophyletic lineages. However, support values for the split between SFTPC and SFTPC-like are low, leaving a polytomy including lungfish SFTPC, coelacanth SFTPC, salamander SFTPC-like and tetrapod SFTPC (Figs. S1, S2). Bayesian (Fig. S1b) and maximum likelihood (Fig. S2) gene trees both place coelacanth SFTPC sister to salamander SFTPC-like, but support values for a sister relationship to salamander SFTPC-like are low under the maximum likelihood approach. The presence of SFTPC in all amphibian and amniote groups and the absence of SFTPC-like in all surveyed tetrapods except salamanders argues that the most parsimonious explanation for the corresponding gene tree is that the tetrapod ortholog of SFTPC was duplicated in the salamander lineage following its divergence from frogs, followed by substantial sequence divergence between SFTPC and SFTPC-like (Fig. S1b). Low statistical support at the salamander SFTPC-like + coelacanth SFTPC node under maximum likelihood approaches should be interpreted as a polytomy, and long-branch attraction may cause an artifactual affiliation between coelacanth and lungfish SFTPC orthologs and salamander SFTPC-like. Therefore, origin of SFTPC-like from a salamander-specific gene duplication event is the most parsimonious explanation for the observed gene trees. However, alternative scenarios such as independent losses of SFTPC-like across lungfish, frogs and amniotes cannot be ruled out based purely on gene tree topology from Bayesian and maximum likelihood approaches alone.

Sequence alignments alone may not provide sufficient information to reconstruct gene evolution and patterns of gene gain and loss [16]. We applied PHYLDOG [16] to explicitly test for gene duplications of SFTPC. Given a guide tree with known phylogenetic relationships (Fig. S3), PHYLDOG predicts that SFTPC-like arose by gene duplication after the divergence of frogs and salamanders (Fig. S4). SFTPC-like has been meiotically mapped to linkage group 6 in *Ambystoma*

mexicanum, a lunged salamander, and is located within a region syntenic to human chromosome 15 [17]. Finally, SFTPC-like and SFTPC have been assembled to two separate genomic scaffolds from *A. mexicanum* [18], supporting SFTPC-like's origin via gene duplication.

Previous work identified a SFTPC gene duplication event in the lungfish *Protopterus annectens* [19]. Our analysis confirms that this duplication event is lineage specific and confined to *P. annectens* (Fig. S1b).

Supporting Information Tables:

Table S1. Primers used to clone SFTPC and SFTPC-like from *Ambystoma mexicanum, Desmognathus fuscus* and *Plethodon cinereus*.

Gene	Species	Forward	Reverse
SFTPC	*A. mexicanum*	5'-CAC ACA GAR AMG ATT TTC CAG ATG-3'	5'-CGT CTT GTC CAT TTT TGT KAB GTA GCA-3'
SFTPC-like	*A. mexicanum*	5'-AAG ATG GAA ACC GGC AGC AAG C-3'	5'-CGT CTT GTC CAT TTT TGT KAB GTA GCA-3'
SFTPC-like	*D. fuscus*	5'-AAG ATG GAA ACC GGC AGC AAG C-3'	5'-AGT ATT GGA AGC GGT CTG GGT G-3'
SFTPC-like	*P. cinereus*	5'-AAG ATG GAA ACC GGC AGC AAG C-3'	5'-GGT GTA GTC ATA GAC CAC-3'

Table S2. Transcriptomes used to identify SFTPC and SFTPC-like.

Accession numbers are listed in Supplemental Data File 1.

**Species**	**Transcriptome Source**
**Salamanders**	
Ambystoma andersoni	Ryan Woodcock and Randal Voss
Ambystoma mexicanum	Present study
Ambystoma tigrinum	Ryan Woodcock and Randal Voss
Ambystoma tigrinum	[20]
Andrias davidianus	[21]
Cryptobranchus alleganiensis bishopi	David Weisrock and Paul Hime
Cynops cyanurus	David Weisrock and Paul Hime
Desmognathus fuscus	David Weisrock and Justin Kratovil
Ensatina eschscholtzii	Rachel Mueller [22]
Hynobius chinensis	[23], reassembled by Paul Hime
Hynobius retardatus	[24]
Notophthalmus viridescens	[25]
Plethodon cinereus	Present study
**Frogs**	
Pelophylax nigromaculatus	[26]
Rana (Lithobates) pipiens	[27]
**Dipnoi**	
Lepidosiren paradoxa	Igor Schneider [28]
Protopterus annectens	Chris Amemiya [28]; [19]

Supporting Information Figures:

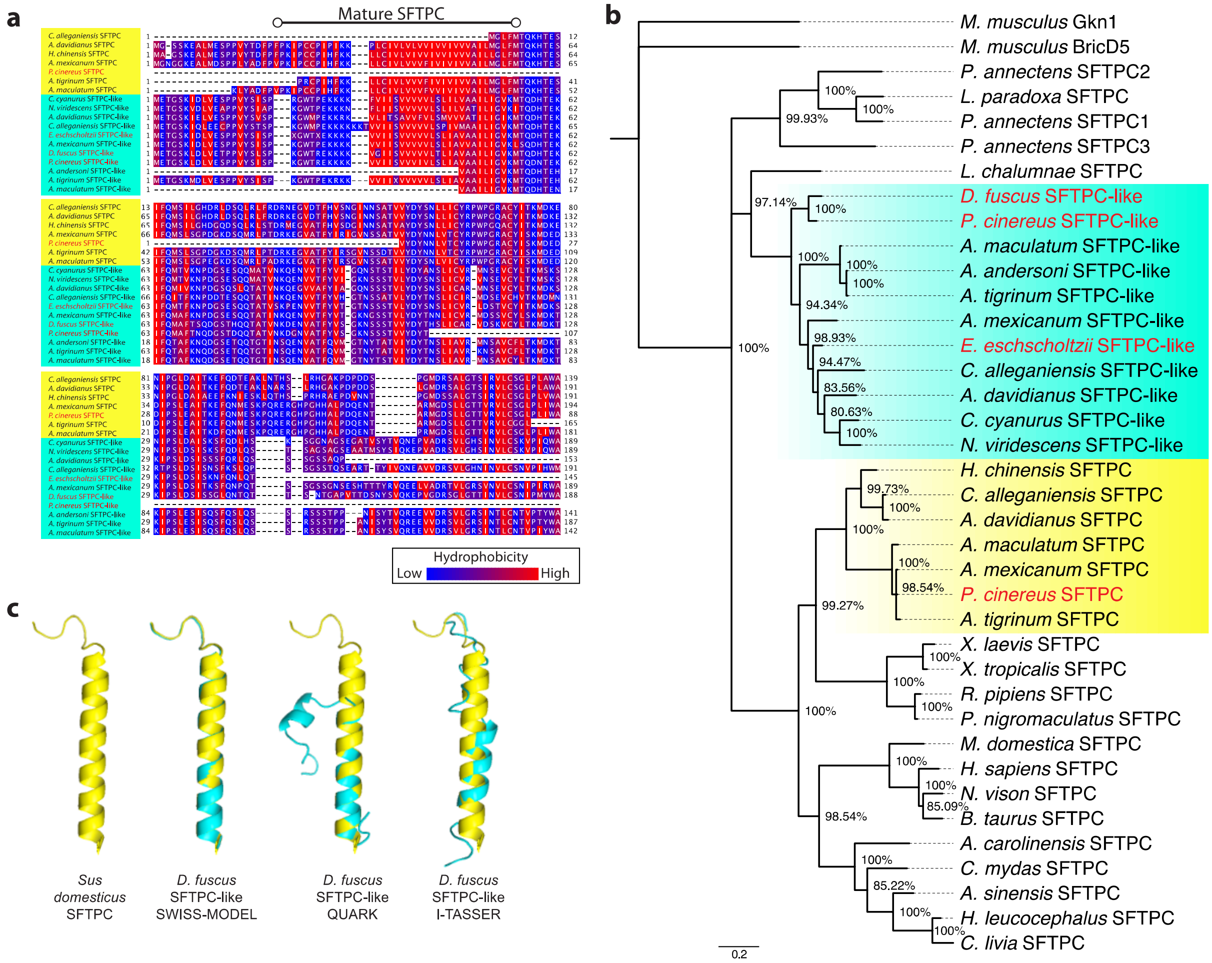

Figure S1. A novel form of Surfactant-associated protein C (SFTPC) is expressed in several species of salamanders. a, Amino acid alignment of SFTPC (yellow) and SFTPC-like (cyan) sequences reveals conservation of hydrophobic residues within the mature peptide domain. Full species names and accession numbers are listed in Supplemental Data File 1. Lungless (plethodontid) species are in red font. **b,** Bayesian 95% maximum clade credibility tree for SFTPC reveals SFTPC-like transcripts in 11 species of salamanders. SFTPC-like is not a related ortholog because it is nested within the SFTPC phylogeny. Node values are posterior probabilities; scale bar denotes expected changes per site. Salamander SFTPC is marked with a yellow box; salamander SFTPC-like is marked in cyan. **c,** Predicted secondary structure of SFTPC-like from *Desmognathus fuscus*. SFTPC-like structure predictions (cyan) utilizing SWISS-MODEL [29], QUARK *Ab initio* predictions [30] and I-TASSER [31] are aligned with the resolved SFTPC mature peptide (yellow) [32].

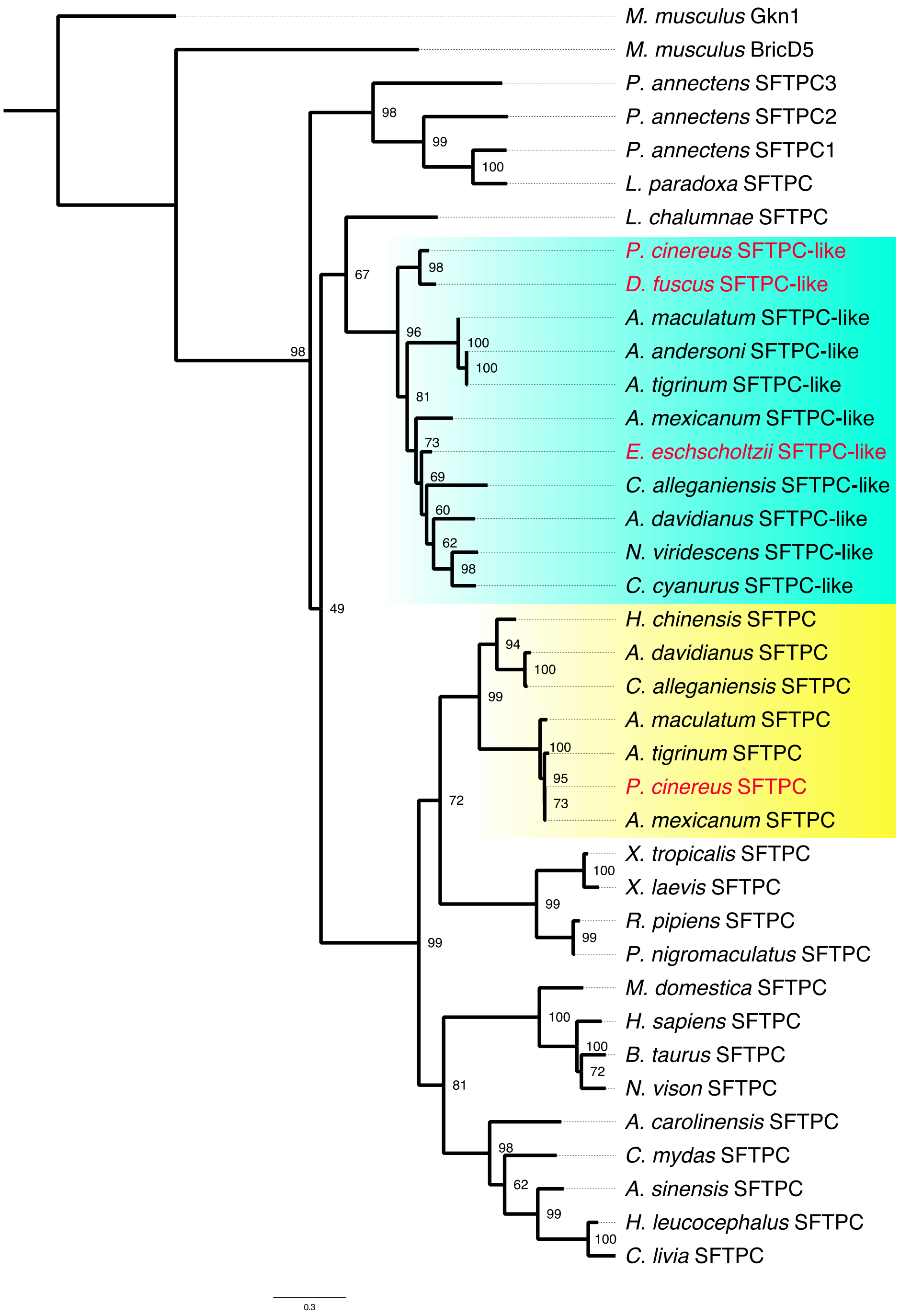

Figure S2. Maximum likelihood gene tree for SFTPC and SFTPC-like. RAxML (v8.2.10) tree with 1000 bootstrap inferences and the JTT substitution matrix. Bootstrap support values from the maximum likelihood bootstraps are labeled on each node. Monophyly of coelacanth (*Latimeria chalumnae*) SFTPC + salamander SFTPC-like (cyan box) is weakly supported. Salamander SFTPC is marked with a yellow box. Full species names and accession numbers are listed in Supplemental Data File 1. Lungless (plethodontid) species are in red font.

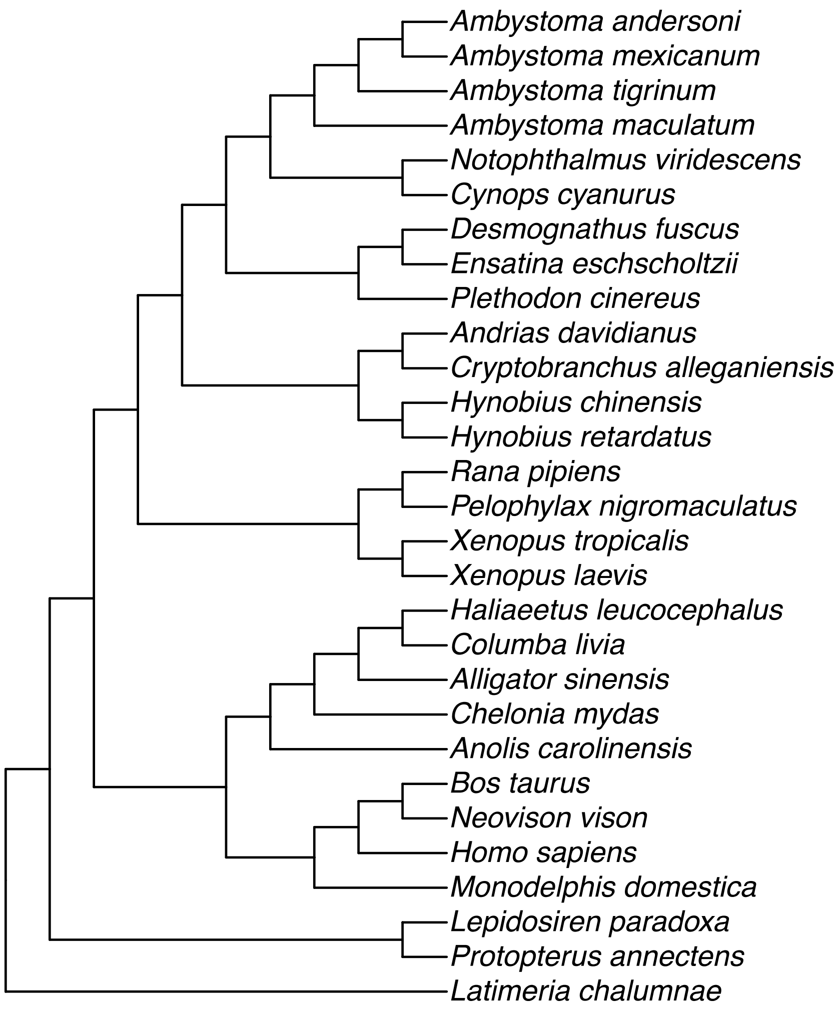

Figure S3. Guide tree for PHYLDOG. The NCBI taxonomic database was used to generate the tree topology, combined with Pyron and Wiens (2011) [33] for amphibian phylogenetic relationships.

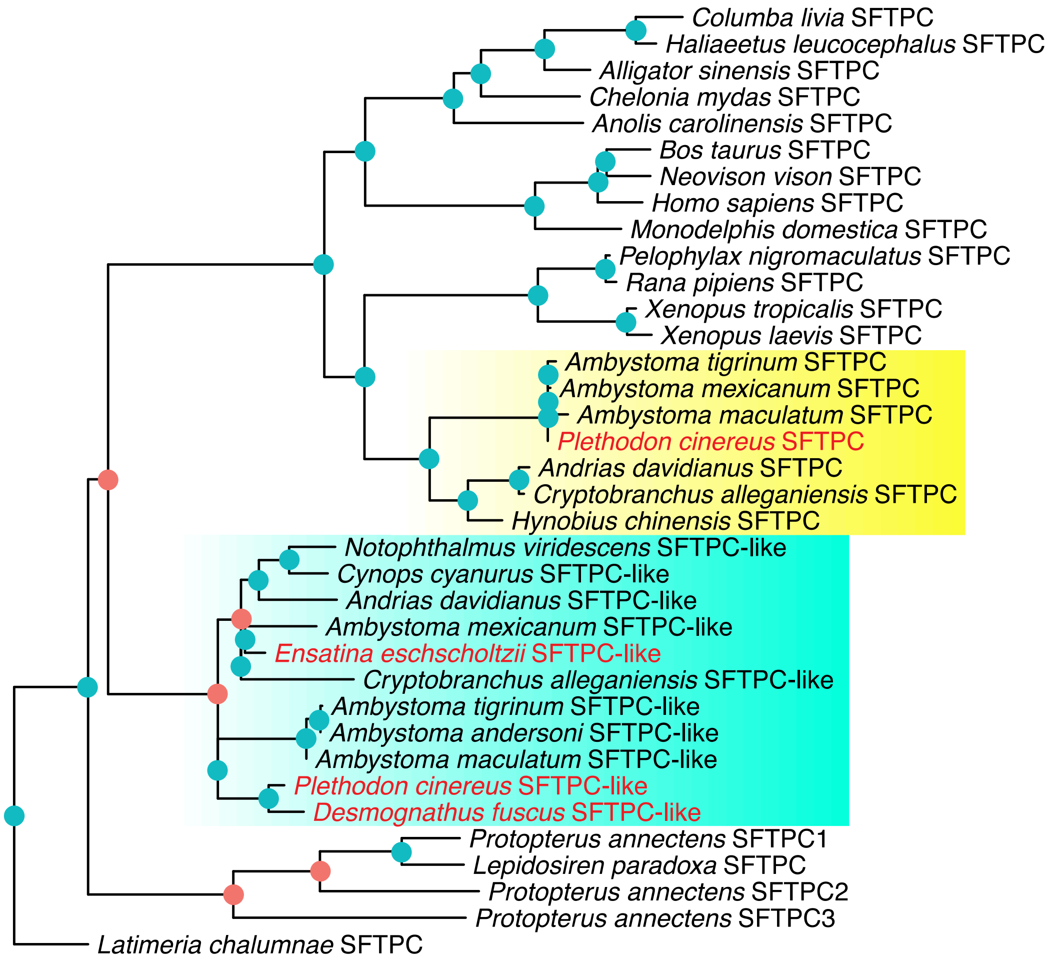

Figure S4. Gene duplications predicted by PHYLDOG. The nodes where a gene duplication event is predicted are colored red. Blue nodes indicate divergence due to speciation events. PHYLDOG predicts that SFTPC-like (cyan box) originated due to gene duplication. Two other duplication events of SFTPC-like are predicted in salamanders. While some species of salamanders appear to express only one form of SFTPC-like, several species express SFTPC-like transcripts with slight sequence differences (Supplemental Data File 1). Further work is needed to determine if these sequence differences indeed represent further duplications of SFTPC-like or instead are the result of assembly error or alternative splicing. Only one SFTPC-like sequence per species was selected for phylogenetic analysis. Salamander SFTPC is marked with a yellow box. Lungless (plethodontid) species are in red font.

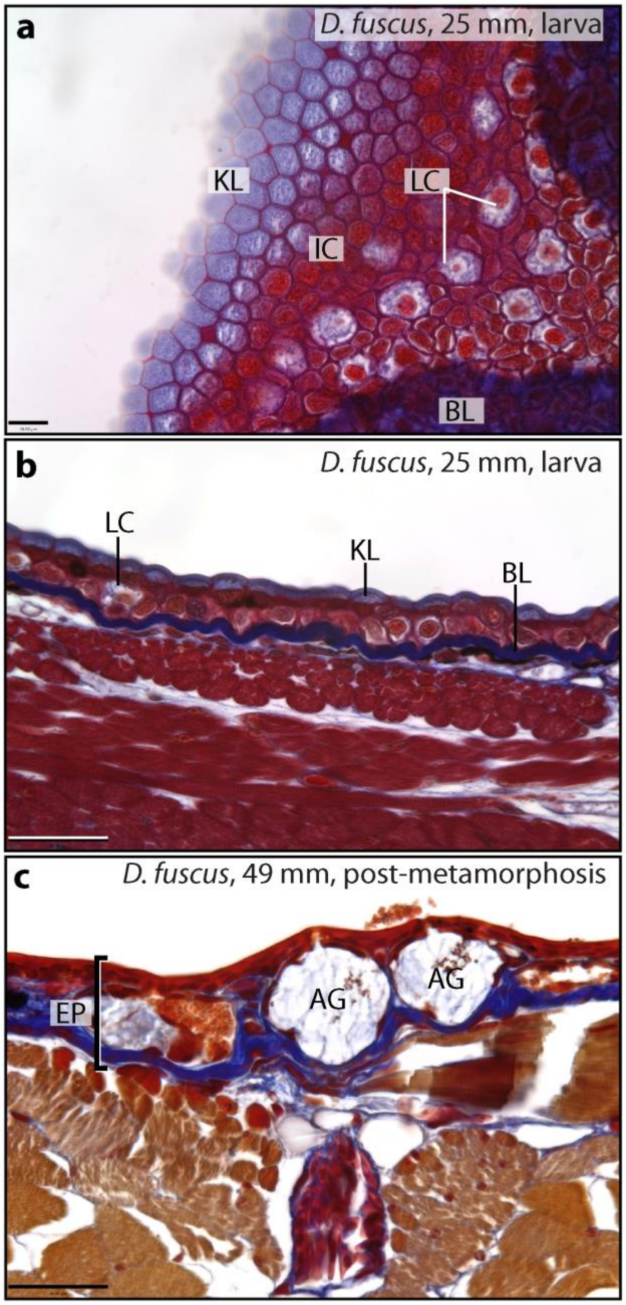

Figure S5. Histology of the integument in *Desmognathus fuscus* before and after metamorphosis. a, Tangential section through the gular region of a larva shows the layers of the integument (from left to right): flattened, cuticle-like keratinized layer (KL); an inner cell layer (IC); large cuboidal Leydig cells (LC) intermingled with capillaries and other supporting cells; basal lamina (BL). **b,** Sagittal section from the abdominal region of a larva. **c,** Transverse section from a recently metamorphosed specimen showing the acinous glands (AG) and a greatly thickened epidermis (EP). Mallory trichrome stain. Scale bars: **a**, 20 µm; **b,c,** 50 µm.

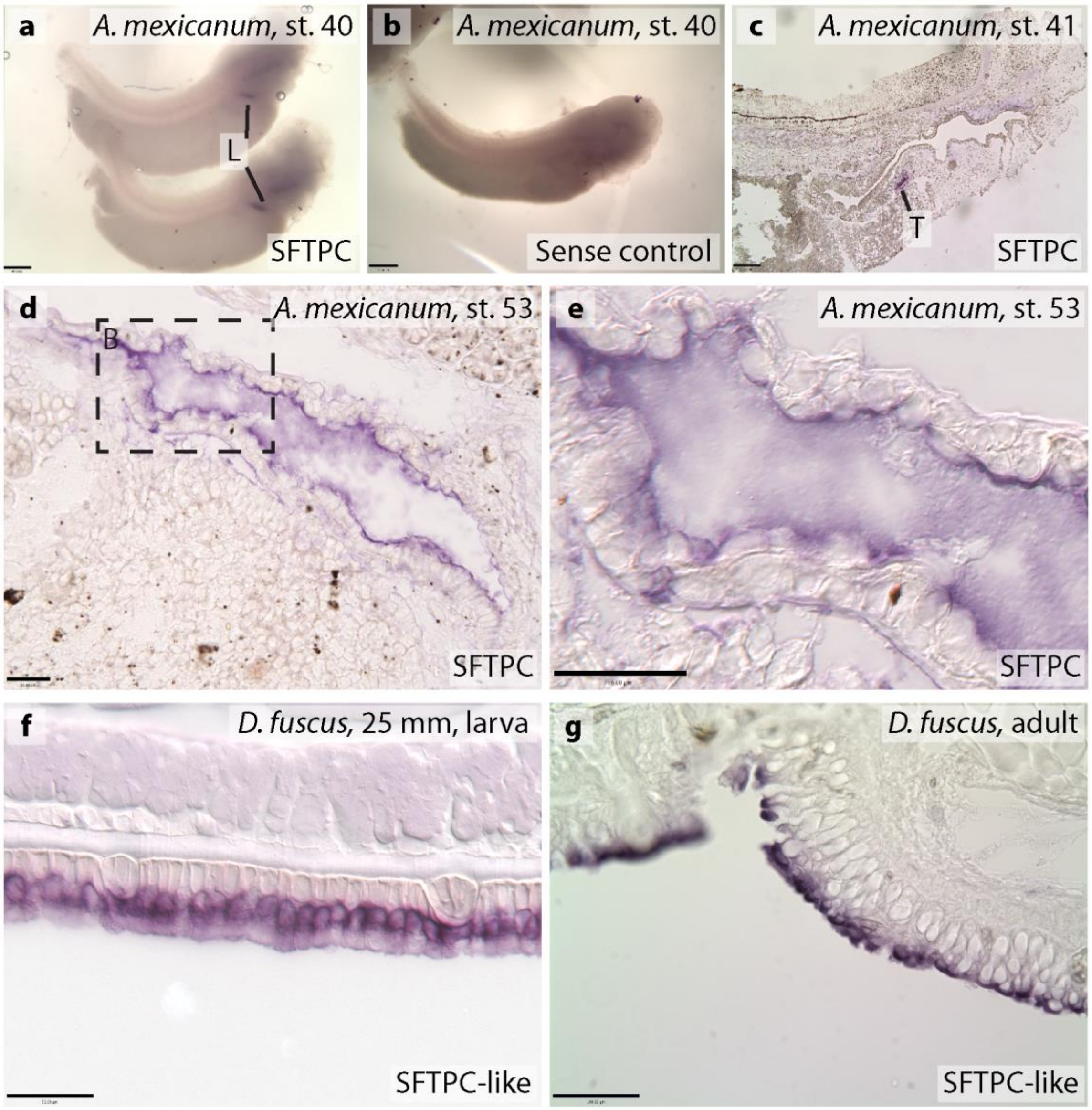

Figure S6. Additional images of SFTPC and SFTPC-like expression patterns. a, Wholemount embryos of *Ambystoma mexicanum* display SFTPC expression specific to the lungs (L). Lateral view; anterior is to the right. **b**, SFTPC sense control at the same stage shows no lung expression. **c,** Midsagittal section of *A. mexicanum* embryo stained for SFTPC shows expression in the trachea (T), but no expression in the integument or buccopharynx. Dorsal is up, anterior is to the right. **d,e,** SFTPC expression in *A. mexicanum* lung is confined to squamous epithelial cells lining the lumen. Sagittal section with anterior to the right. **e** is an enlargement of the boxed region in **d**. **f,** SFTPC-like expression in *Desmognathus fuscus* integument is confined to the apical cellular layer. Sagittal section with anterior to the left. **g,** SFTPC-like expression in adult *D. fuscus* buccal epithelium. Transverse section. Scale bars: **d,e,f,** 50 µm; **a,b,c,g,** 100 µm.

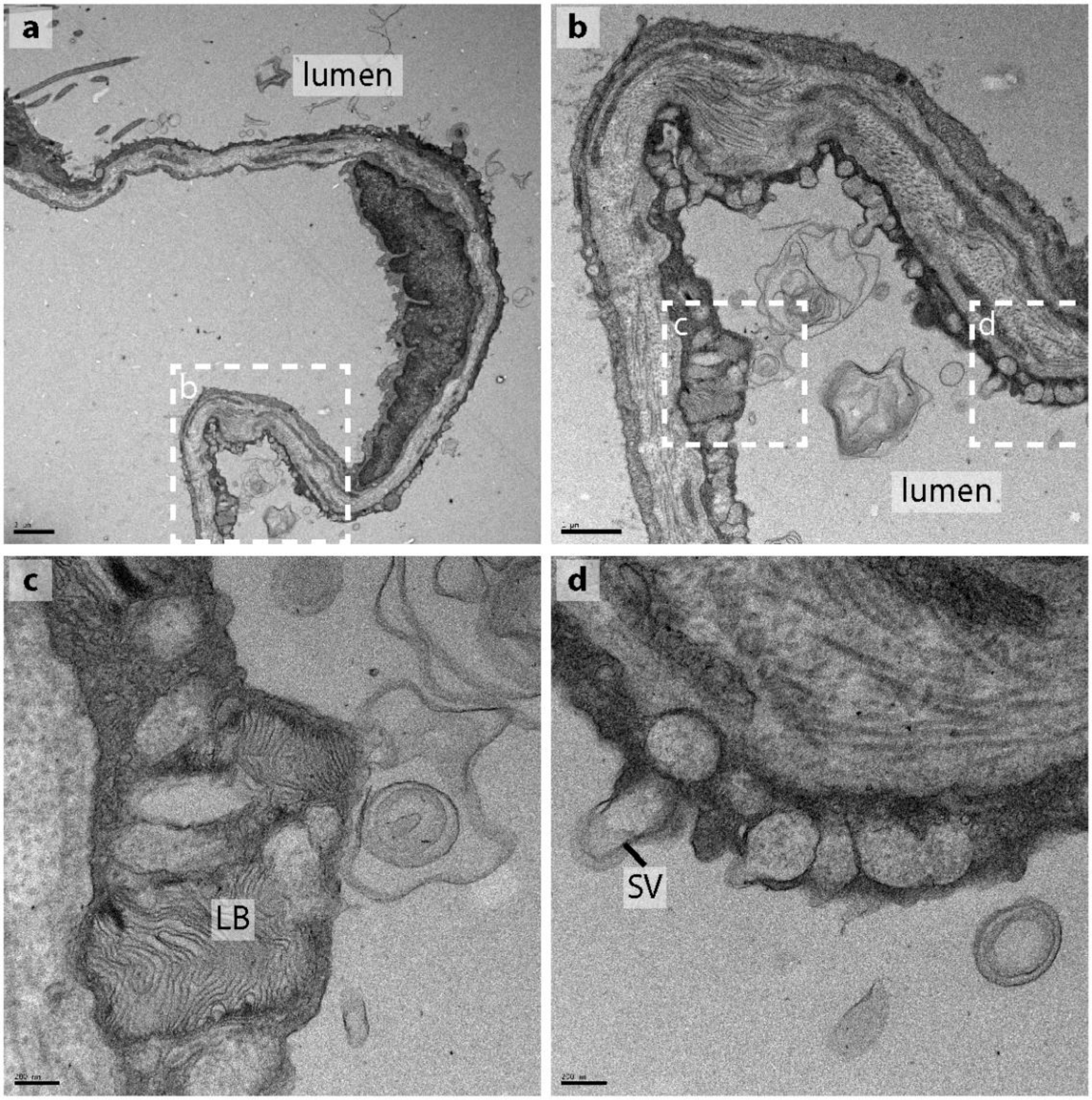

Figure S7. Ultrastructure of alveolar epithelial cells in adult *Ambystoma mexicanum*. a, Low magnification view of the pulmonary epithelium. The lumen of the lung is to the right. **b,** Enlargement of boxed area in **a**. **c,d,** Enlargement of boxed regions in **b** show lamellar bodies (LB) and secretory vesicles (SV). Scale bars: **a,** 2 µm; **b,** 1 µm; **c,d,** 200 nm.

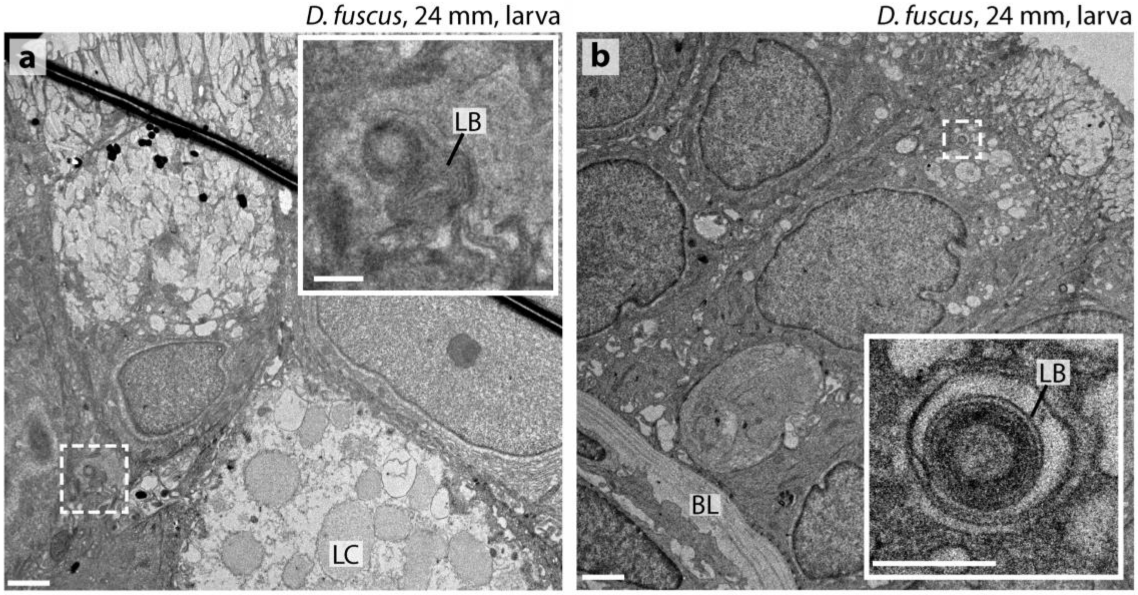

Figure S8. Lamellar bodies in *Desmognathus fuscus* integument. a, b, Additional examples of lamellar bodies (LB) found in skin from a 24-mm larva of *D. fuscus,* a lungless salamander. The lamellar bodies are large (> 0.5 µm) and localized to the apical layer of epidermis. Each inset magnifies its corresponding boxed region (dashed lines). Apical is up in **a** and towards the upper right in **b**. Abbreviations: BL, basal lamina; LC, Leydig cell. Scale bars: **a,b,** 2 µm (insets: 500 µm).

Captions for Supplemental Data Files:

Supplemental Data File 1: Excel spreadsheet with all sequence data used for the study.
Supplemental Data File 2: A FASTA amino acid alignment used to generate the gene tree.

Supporting Information References:

1. Bourbon JR, Chailley-Heu B. 2001 Surfactant proteins in the digestive tract, mesentery, and other organs: evolutionary significance. *Comp. Biochem. Physiol. A, Mol. Integr. Physiol.* **129**, 151–161.
2. Fisher JH, Shannon JM, Hofmann T, Mason RJ. 1989 Nucleotide and deduced amino acid sequence of the hydrophobic surfactant protein SP-C from rat: expression in alveolar type II cells and homology with SP-C from other species. *Biochim. Biophys. Acta* **995**, 225–30.
3. Glasser SW, Burhans MS, Eszterhas SK, Bruno MD, Korfhagen TR. 2000 Human SP-C gene sequences that confer lung epithelium-specific expression in transgenic mice. *Am. J. Physiol. Lung Cell. Mol. Physiol.* **278**, L933-45.
4. Wert SE, Glasser SW, Korfhagen TR, Whitsett JA. 1993 Transcriptional elements from the human SP-C gene direct expression in the primordial respiratory epithelium of transgenic mice. *Dev. Biol.* **156**, 426–443.
5. Rankin SA *et al.* 2015 A molecular atlas of *Xenopus* respiratory system development. *Dev. Dyn.* **244**, 69–85.
6. Hyatt BA, Resnik ER, Johnson NS, Lohr JL, Cornfield DN. 2007 Lung specific developmental expression of the *Xenopus laevis* surfactant protein C and B genes. *Gene Expr. Patterns* **7**, 8–14.
7. Weaver TE, Whitsett JA. 1991 Function and regulation of expression of pulmonary surfactant-associated proteins. *Biochem. J.* **273**, 249–264.
8. Wohlford-Lenane CL, Durham PL, Snyder JM. 1992 Localization of Surfactant-associated Protein C (SP-C) mRNA in fetal rabbit lung tissue by in situ hybridization. *Am. J. Respir. Cell Mol. Biol.* **6**, 225–234.
9. Yin A, Winata CL, Korzh S, Korzh V, Gong Z. 2010 Expression of components of Wnt and Hedgehog pathways in different tissue layers during lung development in *Xenopus laevis*. *Gene Expr. Patterns* **10**, 338–44.
10. Rankin SA, Gallas AL, Neto A, Gómez-Skarmeta JL, Zorn AM. 2012 Suppression of Bmp4 signaling by the zinc-finger repressors Osr1 and Osr2 is required for Wnt/β-catenin-mediated lung specification in *Xenopus*. *Development* **139**, 3010–20.
11. Mo YK, Kankavi O, Masci PP, Mellick GD, Whitehouse MW, Boyle GM, Parsons PG, Roberts MS, Cross SE. 2006 Surfactant protein expression in human skin: evidence and implications. *J. Invest. Dermatol.* **127**, 381–386.
12. Bräuer L, Möschter S, Beileke S, Jäger K, Garreis F, Paulsen FP. 2009 Human parotid and submandibular glands express and secrete surfactant proteins A, B, C and D. *Histochem. Cell Biol.* **132**, 331–8.

13. Schicht M *et al.* 2015 The distribution of human surfactant protein within the oral cavity and their role during infectious disease of the gingiva. *Ann. Anat.* **199**, 92–7.
14. Beers MF, Mulugeta S. 2005 Surfactant protein C biosynthesis and its emerging role in conformational lung disease. *Annu. Rev. Physiol.* **67**, 663–696.
15. Schob S, Schicht M, Sel S, Stiller D, Kekulé A, Paulsen F, Maronde E, Bräuer L. 2013 The detection of surfactant proteins A, B, C and D in the human brain and their regulation in cerebral infarction, autoimmune conditions and infections of the CNS. *PLoS One* **8**, e74412.
16. Boussau B, Szollosi GJ, Duret L, Gouy M, Tannier E, Daubin V. 2013 Genome-scale coestimation of species and gene trees. *Genome Res.* **23**, 323–330.
17. Voss SR, Kump DK, Putta S, Pauly N, Reynolds A, Henry RJ, Basa S, Walker JA, Smith JJ. 2011 Origin of amphibian and avian chromosomes by fission, fusion, and retention of ancestral chromosomes. *Genome Res.* **21**, 1306–1312.
18. Nowoshilow S *et al.* 2018 The axolotl genome and the evolution of key tissue formation regulators. *Nature* **554**, 50–55.
19. Biscotti MA, Gerdol M, Canapa A, Forconi M, Olmo E, Pallavicini A, Barucca M, Schartl M. 2016 The lungfish transcriptome: A glimpse into molecular evolution events at the transition from water to land. *Sci. Rep.* **6**, 21571.
20. Doyle JM, Siegmund G, Ruhl JD, Eo SH, Hale MC, Marra NJ, Waser PM, Dewoody JA. 2013 Microsatellite analyses across three diverse vertebrate transcriptomes. *Genome* **56**, 407–414.
21. Su S *et al.* 2018 Comparative expression analysis identifies the respiratory transition-related miRNAs and their target genes in tissues of metamorphosing Chinese giant salamander (*Andrias davidianus*). *BMC Genomics* **19**, 406.
22. Mohlhenrich ER, Mueller RL. 2016 Genetic drift and mutational hazard in the evolution of salamander genomic gigantism. *Evolution.* **70**, 2865–2878.
23. Che R, Sun Y, Wang R, Xu T. 2014 Transcriptomic analysis of endangered Chinese salamander: Identification of immune, sex and reproduction-related genes and genetic markers. *PLoS One* **9**, e87940.
24. Matsunami M, Kitano J, Kishida O, Michimae H, Miura T, Nishimura K. 2015 Transcriptome analysis of predator- and prey-induced phenotypic plasticity in the Hokkaido salamander (*Hynobius retardatus*). *Mol. Ecol.* **24**, 3064–3076.

25. Abdullayev I, Kirkham M, Bjorklund SK, Simon A, Sandberg R. 2013 A reference transcriptome and inferred proteome for the salamander *Notophthalmus viridescens*. *Exp. Cell Res.* **319**, 1187–1197.
26. Huang L, Li J, Anboukaria H, Luo Z, Zhao M, Wu H. 2016 Comparative transcriptome analyses of seven anurans reveal functions and adaptations of amphibian skin. *Sci. Rep.* **6**, 24069.
27. Christenson MK *et al.* 2014 De novo assembly and analysis of the Northern leopard frog *Rana pipiens* transcriptome. *J. Genomics* **2**, 141–9.
28. Amemiya CT *et al.* 2013 The African coelacanth genome provides insights into tetrapod evolution. *Nature* **496**, 311–316.
29. Arnold K, Bordoli L, Kopp J, Schwede T. 2006 The SWISS-MODEL workspace: a web-based environment for protein structure homology modelling. *Bioinformatics* **22**, 195–201.
30. Xu D, Zhang Y. 2012 Ab initio protein structure assembly using continuous structure fragments and optimized knowledge-based force field. *Proteins* **80**, 1715–1735.
31. Yang J, Yan R, Roy A, Xu D, Poisson J, Zhang Y. 2015 The I-TASSER Suite: protein structure and function prediction. *Nat. Methods* **12**, 7–8.
32. Johansson J, Szyperski T, Curstedt T, Wüthrich K. 1994 The NMR structure of the pulmonary surfactant-associated polypeptide SP-C in an apolar solvent contains a valyl-rich alpha-helix. *Biochemistry* **33**, 6015–6023.
33. Pyron RA, Wiens JJ. 2011 A large-scale phylogeny of Amphibia including over 2,800 species, and a revised classification of extant frogs, salamanders, and caecilians. *Mol. Phylogenet. Evol.* **61**, 543–583.
